# Managing Density Stress to Close the Maize Yield Gap

**DOI:** 10.3389/fpls.2021.767465

**Published:** 2021-12-15

**Authors:** Eric T. Winans, Tryston A. Beyrer, Frederick E. Below

**Affiliations:** Department of Crop Sciences, University of Illinois at Urbana-Champaign, Urbana, IL, United States

**Keywords:** maize, density, population, spacing, fertility, nitrogen, yield, kernel

## Abstract

Continued yield increases of maize (*Zea mays* L.) will require higher planting populations, and enhancement of other agronomic inputs could alleviate density-induced stress. Row spacing, plant population, P-S-Zn fertility, K-B fertility, N fertility, and foliar protection were evaluated for their individual and cumulative impacts on the productivity of maize in a maize-soybean [*Glycine max* (L.) Merr.] rotation. An incomplete factorial design with these agronomic factors in both 0.76 and 0.51 m row widths was implemented for 13 trials in Illinois, United States, from 2014 to 2018. The agronomic treatments were compared to two controls: enhanced and standard, comprising all the factors applied at the enhanced or standard level, respectively. The 0.51 m enhanced management control yielded 3.3 Mg ha^–1^ (1.8–4.6 Mg ha^–1^ across the environments) more grain (25%) than the 0.76 m standard management control, demonstrating the apparent yield gap between traditional farm practices and attainable yield through enhanced agronomic management. Narrow rows and the combination of P-S-Zn and K-B fertility were the factors that provided the most significant yield increases over the standard control. Increasing plant population from 79,000 to 109,000 plants ha^–1^ reduced the yield gap when all other inputs were applied at the enhanced level. However, increasing plant population alone did not increase yield when no other factors were enhanced. Some agronomic factors, such as narrow rows and availability of plant nutrition, become more critical with increasing plant population when density-induced stress is more significant. Changes in yield were dependent upon changes in kernel number. Kernel weight was the heaviest when all the management factors were applied at the enhanced level while only planting 79,000 plants ha^–1^. Conversely, kernel weight was the lightest when increasing population to 109,000 plants ha^–1^ while all other factors were applied at the standard level. The yield contribution of each factor was generally greater when applied in combination with all other enhanced factors than when added individually to the standard input system. Additionally, the full value of high-input agronomic management was only realized when matched with greater plant density.

## Introduction

Due to breeding advancements and improved crop management practices, substantial gains in maize (*Zea mays* L.) yield in the United States have been made to-date (Duvick, 2005; Lee and Tollenaar, 2007). However, on-farm maize yields are estimated to be only 65% of yield potential for the non-irrigated environments typical in the United States (Lobell et al., 2009). This yield gap (the difference between the realized and potential yield) can be lessened with an advanced understanding of the agronomic and genetic factors that influence yield (Dobermann et al., 2002; Ruffo et al., 2015).

Grain yield is the product function of the number of plants per unit area, the number of viable kernels on each plant, and the size of each kernel. Thus, from a physiological perspective, increasing maize yield requires either more kernels per plant or heavier kernels while keeping the plant population constant (i.e., greater yield potential) or the ability to maintain kernel number and weight while increasing the plant population (i.e., greater density tolerance) (Gonzalez et al., 2018). Contemporary maize hybrids have greater yield potential as a direct result of greater crowding-stress tolerance (Tokatlidis and Koutroubas, 2004; Lee and Tollenaar, 2007; Hammer et al., 2009; Gonzalez et al., 2018), which has led to greater within- and between-field variability in grain yield in the United States Corn Belt (Lobell and Azzari, 2017). Currently, maize hybrids are grown at an average population of about 79,000 plants ha^–1^ in the United States Corn Belt, which has increased by approximately 1% annually since the mid-1990s (USDA-NASS, 2021). As plant populations rise, intraspecific competition for limiting resources increases, leading to increased plant-to-plant variability (Boomsma et al., 2009) and reduced plant growth and survival (Casper and Jackson, 1997). Several physiological changes, such as decreased root biomass, occur due to increased plant populations, which can lessen the ability of the crop to obtain resources and potentially reduce grain yield (Jiang et al., 2013; Bernhard and Below, 2020). The future of maize yield improvement may need to focus on crop management strategies and hybrid selection that alleviate stresses at higher plant populations (Tollenaar and Lee, 2002).

Reducing row spacing (<0.76 m) increases plant-to-plant spacing within the row and potentially increases yield through better light interception and more efficient usage of available space and resources (Andrade et al., 2002; Sharratt and McWilliams, 2005; Barbieri et al., 2008). The root weight of individual maize plants decreases by 1.2% for every 1,000 plants ha^–1^ increase in population (Bernhard and Below, 2020). However, increasing plant-to-plant spacing within the row by decreasing row spacing from 0.76 to 0.51 m increased root weight by 22%, which improves the plant’s ability to obtain limiting resourses (i.e., water and nutrients) at higher populations. Past research on narrow-row maize (row spacing less than the current average of 0.76 m in the United States) has shown mixed results (Nielson, 1988; Porter et al., 1997; Cox et al., 1998), suggesting geography, hybrid, and other factors may affect the yield response of maize to narrow row spacing.

Nutrient deficiency is the most common yield-limiting factor worldwide for maize (Mueller et al., 2012). Increased plant demand for soil nutrients at higher populations (Ciampitti and Vyn, 2012) and declining soil test levels in the United States Midwest (Fixen et al., 2010) necessitate improved fertilizer application methods to close the maize yield gap. Harvested grain removes more phosphorus (P) from the field than any other nutrient (Bender et al., 2013). However, P is the least soil-available of the major plant nutrients (Kovar and Claasen, 2005) and is the second most yield-limiting nutrient after nitrogen (N) (Andraski and Bundy, 2008). Additionally, since 2005, the median soil P test value of Illinois, United States, has declined (Fixen et al., 2010). Fertilization of immobile nutrients, such as P and potassium (K), is typically accomplished with broadcast applications, spreading fertilizer in an even distribution across the soil surface and incorporation through conventional tillage. An alternative to broadcast applications is the banding of P and K (i.e., concentrated band 10–15 cm below the soil surface) before planting, which can potentially reduce fixation, increase P and K soil test levels near the root zone, and increase nutrient uptake (Boomsma et al., 2007). Nitrogen, behind carbon, is the mineral nutrient required in the most significant quantities by plants (Hawkesford et al., 2012; Bender et al., 2013), explaining why N fertilizer demand for crop production in North America was approximately 14.5 million tons in 2019 (Food and Agriculture Organization [FAO], 2019). However, applied N that is in excess or unused by the crop is subject to loss and can result in environmental pollution (Dinnes et al., 2002). Practices, such as split applications of N fertilizer or the use of urease and nitrifications inhibitors, can synchronize N availability with crop need and limit losses to the environment (Dinnes et al., 2002; Fageria and Baligar, 2005). Sidedress N applications to maize can be especially practical at increasing grain yield at higher plant populations (Ciampitti and Vyn, 2011). Sulfur (S) deficiency is more frequent than any other secondary nutrient in the United States Corn Belt primarily due to the reduced atmospheric deposition resulting from more rigorous emission standards and rising removal rates by higher grain yields (Lynch et al., 2000; Camberato and Casteel, 2010; Sawyer et al., 2012). Sulfur is the secondary nutrient with the largest harvest index for maize and has season-long uptake (Bender et al., 2013). Zinc (Zn) is the micronutrient most commonly and severely limiting maize yield (Bell and Dell, 2008; Alloway, 2009). Furthermore, Zn is the micronutrient with the highest harvest index in maize (Bender et al., 2013).

A class of systemic fungicides called quinone-outside inhibitors, also referred to as strobilurin fungicides, can be effective against common fungal pathogens that hybrid maize is susceptible to Grossmann and Retzlaff (1997). However, research has shown that they can increase maize yields even when the fungal diseases are not detectable in the crop (Ruffo et al., 2015). These strobilurin fungicides can have a “greening effect,” resulting in increased photosynthetic capacity and reduced respiration (Grossmann et al., 1999; Bartlett et al., 2002).

Further increasing maize yields necessitates greater planting populations. A clearer knowledge of which agronomic management practices have the most significant impact on maize yield and how these practices interact with increased density is needed. Therefore, this research aimed to demonstrate the potential for improved maize productivity *via* increased planting populations and enhanced crop management and to evaluate the individual and synergistic contributions of soil fertility, supplemental nitrogen, planting population, foliar protection, and row spacing on grain yield and yield components.

## Materials and Methods

In this research, 13 field trials were conducted in different environments during the 2014–2018 growing seasons at the Crop Sciences Research and Education Center in Champaign-Urbana (CU) (40°2′ N, 88°14′ W) in east-central Illinois and the Northern Illinois Agronomy Research Center near DeKalb (DK) (41°47′ N, 88°50′ W) in northern Illinois, United States. The fields used at each site were located within 1 km of each other and had similar soil types, fertility levels, and management histories. Soybean was the previous crop, and tillage practices were generally classified as conventional deep ripping followed by cultivation tillage at each field site. An average of two trials was established in each environment and differed in their maize hybrid and plant protection products. The number of trials in each environment, planting dates, and average soil properties are outlined in Table 1. A complete list of trials, hybrids and foliar protection products used, and soil properties are shown in Supplementary Table 1. All the hybrids planted in this study were commercially available and widely grown in Illinois, United States. Soil samples were taken from a depth of 0 to 15 cm from each trial area before planting, and the minerals were extracted and determined using Mehlich III solution (A&L Great Lakes Laboratories, Fort Wayne, IN, United States). The CU trials were located on soils classified as Flannagan silt loam (fine, smectitic, mesic Aquic Argiudolls) with 0–2% slope and had medium to high levels of P based on the spring soil tests. Research plots near DK were located on soils classified as Drummer silty clay loam (fine-silty, mixed, superactive, mesic Typic Endoaquolls; 0–2% slope), with higher organic matter levels than the soils in CU.

**TABLE 1:** Summary of trial information and soil properties for six environments at Champaign-Urbana (CU) or DeKalb (DK), IL from 2014–2018.

Environment	Total trials	Planting dates	CEC^†^	pH	OM	P	K	Ca	Mg	S	Zn	B
			**meq 100g^–1^**		**%**	**——————————————————————————— ppm —————————————————————————**						
14CU	3	03–06 June 2014	17.9	5.4	3.4	42	133	1832	387	9	1.1	0.3
15CU	2	07–13 May 2015	23.1	5.6	4.0	12	112	2653	569	-	-	-
15DK	1	22 May 2015	27.3	6.7	6.5	42	172	3567	897	8	4.1	-
16CU	3	19–22 April 2016	18.7	6.0	3.3	34	127	2220	487	8	1.6	0.3
17CU	2	18 May 2017	20.7	5.5	3.9	15	100	2321	412	9	1.2	0.4
18CU	2	26 May 2018	19.8	6.4	3.5	38	128	2527	532	9	2.0	0.5

*^†^CEC, cation exchange capacity; OM, organic matter.*

The trials were planted in a randomized complete block design with six replications and two row widths (0.51 and 0.76 m) in a split-plot arrangement. The main-plot was row spacing, and the split-plot was agronomic treatment level. The experimental plots were four rows wide spaced 0.51 or 0.76 m apart and 11.4 m long. The plots were planted with a research plot planter (ALMACO, Nevada, IA, United States) with variable seeding rate capability. Planting dates ranged from late April to early June for all the trials and were reflective of typical planting dates for the region (Table 1). At planting, tefluthrin [(2,3,5,6-tetrafluoro-4-methylphenyl)methyl-(1α,3α)-(Z)-(±)-3-(2-chloro-3,3,3-tri-fluoro-1-propenyl)-2,2-dimethylcyclopropanecarboxylate] was applied in-furrow at a rate of 0.11 kg a.i. ha^–1^ for control of seedling insect pests. Weed control consisted of a pre-emergence application of S-metolachlor [acetamide, 2-chloro-N-(2-ethyl-6-methylphenyl)-N-(2-methoxy-1-methylethyl)-,(S)], atrazine (2-chloro-4-ethylamino-6-isopropylamino-s-triazine), mesotrione {2-[4-(methylsulfonyl)-2-nitrobenzoyl]-1,3-cyclohexanedione}, and bicyclopyrone {bicyclo[3.2.1]oct-3-en-2-one, 4-hydroxy-3-[[2-[(2-methoxyethoxy)methyl]-6-(trifluoromethyl)-3-pyri- dinyl]carbonyl]-} and a post-emergence application of glyphosate [*N*-(phosphonomethyl)glycine].

The center two rows of each plot were mechanically harvested for determining crop grain weight and moisture. The grain yield was calculated based on 15.5% moisture content. The average individual kernel weight was estimated by randomly selecting 300 kernels from each plot and expressed at 0% moisture. Kernel number was estimated by dividing the total plot grain weight by the average individual kernel weight.

### Agronomic Practices

Five management factors were implemented at two levels representing either the “Standard” or “Enhanced” system in 0.76 and 0.51 m row spacings for determining their individual and combined impacts on grain yield (Table 2). The five agronomic management factors considered were: (i) plant fertility to include P, S, and Zn; (ii) K and B fertility; (iii) N fertility; (iv) plant population; and (v) foliar protection.

**TABLE 2:** Addition and omission treatment structure: the treatment exceptions are either added (+factor) to the standard system control or omitted (-factor) from the enhanced system control.

Treatment		Factor				
System	Exception	P-S-Zn	K-B	Nitrogen	Population	Protection
Standard	None^†^	None	None	Base	79,000	None
Standard	+P-S-Zn	P-S-Zn	None	Base	79,000	None
Standard	+K-B	None	K-B	Base	79,000	None
Standard	+P-S-Zn and K-B	P-S-Zn	K-B	Base	79,000	None
Standard	+N	None	None	Base + Sidedress	79,000	None
Standard	+Population	None	None	Base	109,000	None
Standard	+Protection	None	None	Base	79,000	Yes
Enhanced	None	P-S-Zn	K-B	Base + Sidedress	109,000	Yes
Enhanced	−P-S-Zn	None	K-B	Base + Sidedress	109,000	Yes
Enhanced	−K-B	P-S-Zn	None	Base + Sidedress	109,000	Yes
Enhanced	−P-S-Zn and K-B	None	None	Base + Sidedress	109,000	Yes
Enhanced	−N	P-S-Zn	K-B	Base	109,000	Yes
Enhanced	−Population	P-S-Zn	K-B	Base + Sidedress	79,000	Yes
Enhanced	−Protection	P-S-Zn	K-B	Base + Sidedress	109,000	None

*^†^“None” in the exception column indicates the control.*

The value of P-S-Zn and K-B containing fertilizers were tested separately and in combination. The treatment levels for P-S-Zn fertility were none or with added P, S, and Zn denoted as −P-S-Zn or +P-S-Zn, respectively. Immediately before planting, P, S, and Zn were applied as MicroEssentials SZ [12-40-0-10(S)-1(Zn)] (The Mosaic Company, Tampa, FL, United States) in a subsurface band 10–15 cm beneath the future crop row for 34 kg N, 112 kg P_2_O_5_, 28 kg S, and 2.6 kg Zn ha^–1^. Similarly, the two levels for K-B fertility were none or with added K and B, denoted as −K-B or +K-B, respectively. K and B were applied as Aspire [0-0-58-0.5(B)] (the Mosaic Company, Tampa, FL, United States) broadcasted across the soil surface with light incorporation immediately before planting for 84 kg K_2_O and 0.7 kg B ha^–1^ in the enhanced system. In addition, the first two factors were combined with the standard plots receiving no added fertility and the enhanced plots receiving added P-S-Zn and K-B fertility, denoted as −P-S-Zn and K-B or +P-S-Zn and K-B. The −P-S-Zn and K-B would be the typical practice in most fields of this study since the soil test results for P and K were typically above the critical threshold (Culman et al., 2020).

The two levels for the N factor were application at the base rate or base application plus sidedressing, denoted as −N or +N, respectively. For the −N treatment, N was broadcast applied before planting in the spring as 28% urea-ammonium nitrate [UAN, CO(NH_2_)_2_ + NH_4_NO_3_ + H_2_O; 28-0-0] for 180 kg N ha^–1^. The +N treatment received an additional 90 kg N ha^–1^ sidedress at the V6 growth stage as urea with a urease inhibitor [CO(NH2)2 + N-(n-butyl) thiophosphoric triamide; 46-0-0] (BASF Corporation, Research Triangle Park, NC, United States).

Maize was planted for target populations of 79,000 or 109,000 plants ha^–1^, representing a common and high population, denoted as −Pop and +Pop, respectively.

Foliar protection evaluation consisted primarily of a prophylactic fungicide application, but the source of fungicide and tank mixes varied depending on the trial. The applications were made once tassels emerged (plant growth stage VT/R1) using a pressurized CO_2_ back-pack sprayer. The center two rows of each plot were treated with a spray volume of 140 L ha^–1^. The trials received either the fungicide Headline AMP (13.64% Pyraclostrobin + 5.14% Metconazole; 1.05 L ha^–1^; BASF Corporation, Research Triangle Park, NC, United States), the fungicide Quilt Xcel (13.5% Azoxystrobin + 11.7% Propiconazole; 1.05 L ha^–1^; Syngenta Crop Protection, LLC, Greensboro, NC, United States), or the combination of the fungicide Trivapro (10.27% benzovindiflupyr + 10.5% azoxystrobin + 11.9% propiconazole; 1.07 L ha^–1^; Syngenta Crop Protection, LLC, Greensboro, NC, United States) and insecticide Warrior II [22.8% Lambda-cyhalothrin (synthetic pyrethroid); 0.12 L ha^–1^; Syngenta Crop Protection, LLC, Greensboro, NC, United States]. These applications were collectively named “foliar protection” and denoted as +Protection in the enhanced management system to simplify data analysis. In contrast, the standard system received no fungicide application, denoted as −Protection.

### Addition Versus Omission Treatment Structure

The addition versus omission treatment structure used in this study assessed the individual and combined effects of different management factors, resulting in 14 treatments (Table 2). Six addition treatments (+P-S-Zn, +K-B, +P-S-Zn and K-B, +N, +Population, and +Protection) were established by individually substituting the enhanced level of each management factor while all the other management factors remained at the standard level. For example, the +Population treatment was created by substituting 109,000 plants ha^–1^ for 79,000 plants ha^–1^ while all the other management factors remained at the standard level. Similarly, six omission treatments (−P-S-Zn, −K-B, −P-S-Zn and K-B, −N, −Population, and −Protection) were individually substituted for the lower factor level while maintaining all the other factors at the enhanced level. Thus, the −Population treatment was created by substituting the lower plant population (79,000 plants ha^–1^) for the higher plant population (109,000 plants ha^–1^) while all the other management factors were maintained at the enhanced level. In this way, the value of each management factor was tested at the standard level of agronomic management and in an enhanced management system.

### Statistical Analysis

Grain yield and yield components were analyzed with a linear mixed model using the MIXED procedure of SAS version 9.4 (SAS Institute, 2019). Environment (*n=6*), row spacing (*n=2*), agronomic management level (*n=14*), and their interactions were considered to be fixed effects, while trial and replication nested within environment and trial were included in the model as random effects. The normality and homogeneity of the residuals was tested using the Shapiro–Wilks and Brown-Forsythe tests. *T*-tests were used to evaluate the significance of the differences of the least squared means estimates between specific treatments both within and across the row spacings at the 0.1 or 0.05 probability level. The comparisons were comprised of the difference between the enhanced and standard controls, between the six addition treatments (+P-S-Zn, +K-B, +P-S-Zn and K-B, +N, +Population, and +Protection) and the standard control, and between the six omission treatments (−P-S-Zn, −K-B, −P-S-Zn and K-B, −N, −Population, and −Protection) and the enhanced control. Lastly, 95% confidence intervals were estimated for the differences between the enhanced and standard controls across and within the row spacings.

## Results

### Weather

The weather conditions at 14CU were characterized as below-average temperature and above-average precipitation throughout much of the growing season, including heavy rainfall through June and July (Supplementary Table 2). In 2015, Illinois experienced a warm April and May and cooler than average June, July, and August. The month of May had slightly above average rainfall recorded at both 15DK and 15CU. However, June brought extreme rainfalls, with 15DK and 15CU receiving 73 and 113 mm above normal, respectively. July and August were dry for 15DK and 15CU, with relatively favorable temperatures for pollination and grain-fill. The growing season at 16CU experienced near average temperatures and adequate rainfall throughout the growing season. Furthermore, 17CU and 18CU experienced weather that was conducive to high maize yields. The temperatures were near average except for above-average temperature in May at 18CU. Outside of a wet spring, the rain totals were below average for much of the growing season at 17CU. Minimal moisture stress occurred at 18CU, as precipitation did not drastically deviate from normal.

### Row Spacing, Treatment, and Environment Effects on Grain Yield

**FIGURE 1:**
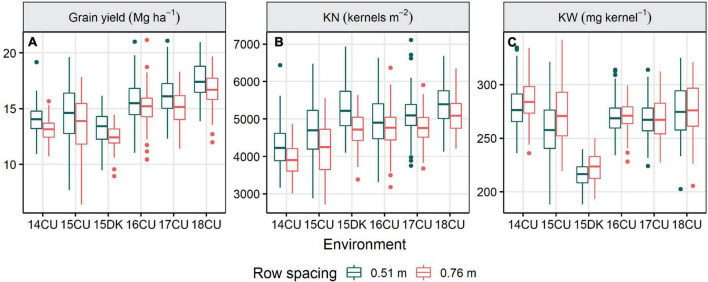
Influence of environment on the grain yield **(A)**, kernel number [KN; **(B)**], and kernel weight [KW; **(C)**] of the 0.51 and 0.76 m row width plots for 14 agronomic treatments, six replications, and an average of two trials in each environment located at Champaign-Urbana (CU) and DeKalb (DK), Illinois from 2014 to 2018. The horizontal lines in the box plot indicate the median. Top and bottom edges of the box refer to the 75th and 25th percentiles, respectively, and whiskers extend to the 10th and 90th percentiles.

Maize grain yield was affected by the environment, row spacing, agronomic treatment, and their interactions (Table 3). Across the six environments, narrowing row spacing from 0.76 to 0.51 m increased yield by 0.6 Mg ha^–1^ (4.5%) in the standard system and 1.2 Mg ha^–1^ (7.8%) in the enhanced system (Table 4), and grain yield was increased from narrowing row spacing at all the environments (Figure 1A). The enhanced management system resulted in a 2.1 and 2.7 Mg ha^–1^ (15.8 and 19.4%) yield increase over the standard control in the wide (0.76 m) and narrow (0.51 m) rows, respectively. Furthermore, the enhanced management system obtained the highest yield in all the environments (Table 5).

**TABLE 3:** ANOVA for maize grain yield (Yield), kernel number (KN), and kernel weight (KW).

Source	Yield	KN	KW
	**——————————————————— *P* > *F* ———————————————————**		
Environment (E)	0.0008	0.0254	0.1513
Row Spacing (S)	< 0.0001	< 0.0001	< 0.0001
E × S	0.0089	< 0.0001	0.0006
Treatment (T)	< 0.0001	< 0.0001	< 0.0001
E × T	< 0.0001	< 0.0001	< 0.0001
S × T	< 0.0001	< 0.0001	0.7271
E × S × T	0.7567	0.4185	0.4663

**TABLE 4:** Maize grain yield (expressed at 15.5% moisture content) response to 14 management systems and the absolute and percentage-wise (in parentheses) difference in yield for the addition or omission treatments relative to the standard or enhanced system controls for two row spacings (0.51 and 0.76 m).

Treatment		0.51 m rows		0.76 m rows	
System	Exception	Yield	Δ	Yield	Δ
		**———————————————— Mg ha^–1^ ———————————————————————**			
Standard	None^†^	13.9		13.3	
Standard	+P-S-Zn	14.6	0.7(5.3%)*	13.9	0.6(4.5%)*
Standard	+K-B	14.1	0.2(1.4%)	13.2	−0.1(−0.8%)
Standard	+P-S-Zn-K-B	14.9	1.0(7.2%)*	14.1	0.8(6.0%)*
Standard	+N	14.6	0.7(5.3%)*	13.9	0.6(4.5%)*
Standard	+Population	13.8	−0.1(−0.7%)	12.9	−0.4(−3.0%)*
Standard	+Protection	14.0	0.1(0.7%)	13.6	0.3(2.3%)‡
Enhanced	None	16.6		15.4	
Enhanced	−P-S-Zn	15.7	−0.9(−5.4%)*	14.6	−0.8(−5.2%)*
Enhanced	−K-B	16.5	−0.1(−0.6%)	14.9	−0.5(−3.2%)*
Enhanced	−P-S-Zn-K-B	15.4	−1.2(−7.2%)*	14.1	−1.3(−8.4%)*
Enhanced	−N	16.0	−0.6(−3.6%)*	14.7	−0.7(−4.5%)*
Enhanced	−Population	15.9	−0.7(−4.2%)*	15.2	−0.2(−1.3%)
Enhanced	−Protection	16.3	−0.3(−1.8%)^‡^	15.1	−0.3(−1.9%)^‡^

Enhanced vs. Standard^§^			2.7(19.4%)*		2.1(15.8%)*

*The values are the average of 13 trials from six environments in Illinois from 2014 to 2018.*

*^†^“None” in the exception column indicates the control.*

*^‡^Significant at the 0.10 probability level compared to the respective control treatment.*

*Significant at the 0.05 probability level compared to the respective control treatment.

*^§^The percentage difference between the standard and enhanced system controls is expressed relative to the standard system control.*

**TABLE 5:** Maize grain yield (expressed at 15.5% moisture content) response to 14 management systems for six environments in Illinois from 2014 to 2018 and the average of environments.

Treatment		Environment						Mean
System	Exception	14CU	15CU	15DK	16CU	17CU	18CU	
		**—————————————————————— Mg ha^–1^ ———————————————————————————**						
Standard	None^†^	12.5	11.9	12.5	14.3	14.6	15.7	13.6
Standard	+P-S-Zn	13.1*	13.4*	12.7	14.8*	15.7*	16.1^‡^	14.3*
Standard	+K-B	12.9^‡^	11.5	12.6	14.1	14.6	16.0	13.6
Standard	+P-S-Zn-K-B	13.2*	14.0*	13.0	14.7^‡^	15.6*	16.5*	14.5*
Standard	+N	13.1*	13.3*	12.9	14.9*	14.9	16.5*	14.3*
Standard	+Population	12.9^‡^	10.9*	11.6*	14.2	14.4	16.1^‡^	13.3*
Standard	+Protection	13.3*	11.6	12.4	14.8*	14.4	16.0	13.8^‡^
Enhanced	None	14.6	15.5	13.5	16.7	17.0	18.6	16.0
Enhanced	−P-S-Zn	14.1*	13.7*	12.9	16.2*	15.6*	18.4	15.2*
Enhanced	−K-B	14.2^‡^	15.8	14.0	16.1*	16.0*	18.0*	15.7*
Enhanced	−P-S-Zn-K-B	14.0*	13.5*	12.6*	16.0*	14.8*	17.6*	14.7*
Enhanced	−N	14.3	14.4*	12.6*	16.2^‡^	16.4*	18.4	15.4*
Enhanced	−Population	13.8*	16.5*	13.9	15.3*	16.4*	17.4*	15.6*
Enhanced	−Protection	13.8*	16.1^‡^	13.4	16.1*	16.5^‡^	18.3	15.7*

*The values are the average of two row spacings (0.76 and 0.51 m) and, on average, two trials within each environment.*

*^†^“None” in the exception column indicates the control.*

*^‡^Significant at the 0.10 probability level compared to the respective control treatment.*

*Significant at the 0.05 probability level compared to the respective control treatment.

### Fertility Effects on Grain Yield

Adding P, S, and Zn fertility to the standard control affected the yield at five of the six environments and, when averaged across all the environments, increased yield by 5% in both row arrangements (Table 4). Also, the omission of P-S-Zn fertility from the enhanced control reduced yield by 0.8 and 0.9 Mg ha^–1^ (5.2 and 5.4%) in wide and narrow rows, respectively. Notably, 15CU and 17CU, the environments with the lowest P soil test levels (Table 1), produced the highest yield responses to P-S-Zn fertility (Table 5). Nonetheless, positive yield responses to P-S-Zn fertilizer were observed in three environments (14CU, 16CU, and 18CU) where soil P levels would be considered adequate.

The potassium and boron fertilizer application did not affect the grain yield when added to the standard management system; however, omitting the K-B fertilizer from the enhanced system when in the wide rows resulted in a 0.5 Mg ha^–1^ (3.2%) yield loss (Table 4).

Removing the combined practices of banded P-S-Zn and broadcast K-B from the enhanced control reduced yield at all the environments (Table 5). Across environments, adding P-S-Zn and K-B fertility to the standard system increased yield by 0.8 Mg ha^–1^ (6.0%) in the wide rows and by 1.0 Mg ha^–1^ (7.2%) in the narrow rows, while their omission from the enhanced system decreased yield by 1.3 Mg ha^–1^ (8.4%) in the wide rows and by 1.2 Mg ha^–1^ (7.2%) in the narrow rows (Table 4). Notably, the yield increases from the individual P-S-Zn and K-B treatments were not additive to the yield response observed when the two treatments were added together, and the P-S-Zn treatment had the most significant contribution to yield response in each management system.

Sidedressing 90 kg N ha^–1^ in addition to the base rate of 180 kg N ha^–1^ in the standard control increased yield in four of the six environments and, on average, yielded an additional 0.7 Mg ha^–1^ (5.1%) over the standard control (Table 5). Additionally, the grain yield was reduced by 0.6 Mg ha^–1^ (3.8%) when the sidedress application was omitted from the enhanced management system.

### Plant Population Effects on Grain Yield

Significant yield increases with the enhanced control over the standard control indicate that the environments tested in this study could support plant populations greater than 79,000 plants ha^–1^ (Table 4). However, increasing plant population from 79,000 to 109,000 plants ha^–1^ in the standard system only increased yield in two environments (14CU and 18CU) and led to yield decreases in two other environments (15CU and 15DK), resulting in a slight average yield decrease (2.2%) (Table 5). The enhanced management system was better able to support the higher density as omitting the high plant population from the enhanced control reduced yield in four of the six environments and, on average, reduced grain yield by 0.4 Mg ha^–1^ (2.5%). The narrower rows were a better arrangement of the high plant population as reducing plant population from 109,000 to 79,000 plants ha^–1^ in the enhanced management system reduced yield by 0.7 Mg ha^–1^ (4.2%) in the 0.51 m spacing and did not affect the yield in the 0.76 m spacing (Table 4). Likewise, increasing the plant population from 79,000 to 109,000 plants ha^–1^ in the standard system only decreased yield in the 0.76 m row spacing while yield was unchanged in the 0.51 m row spacing.

### Foliar Protection Effects on Grain Yield

Measurable fungal leaf infection was not observed in any of the six environments. However, the addition of foliar protection to the standard management control increased yield in two environments (Table 5). In comparison, the omission of foliar protection from the enhanced control affected yield at four environments and, on average, reduced the yield by 0.3 Mg ha^–1^ (1.9%).

### Effects on Yield Components

Environment, row spacing, agronomic treatment, and their interactions strongly affected KN, while KW was affected by row spacing, agronomic treatment, and their interactions with environment (Table 3). Across the treatment levels, switching from 0.76 to 0.51 m row spacing increased KN in all the environments except 16CU and marginally decreased KW in three environments (14CU, 15CU, and 15DK; Figures 1B,C). The difference in KN between the enhanced and standard control treatments (19.3%), when averaged across environment and row spacing, was more significant (*P* < 0.0001) than the observed difference in KW (1.1%; *P* = 0.0098) (Table 6). Additionally, the grain yield was highly correlated with KN (*r* = 0.81,*P* < 0.0001) and less correlated with KW (*r* = 0.22,*P* < 0.0001), suggesting improving KN was more critical than KW for increasing grain yield.

**TABLE 6:** Influence of 14 agronomic management treatments on yield components (kernel number and weight) for two row spacings (0.76 and 0.51 m).

Treatment		Kernel number			Kernel weight		
System	Exception	0.51 m	0.76 m	Mean	0.51 m	0.76 m	Mean
		**———————————— kernels m^–2^ ————————————**			**——————————— mg kernel^–1^ ————————————**		
Standard	None^†^	4,456	4,227	4,342	264	267	265
Standard	+P-S-Zn	4,728*	4,463*	4,595*	263	265	264
Standard	+K-B	4,540	4,146	4,343	263	270^‡^	267
Standard	+P-S-Zn-K-B	4,696*	4,389*	4,542*	269*	273*	271*
Standard	+N	4,641*	4,324^‡^	4,483*	268*	272*	270*
Standard	+Population	4,793*	4,385*	4,589*	245*	249*	247*
Standard	+Protection	4,509	4,267	4,388	263	269	266
Enhanced	None	5,398	4,961	5,180	261	263	262
Enhanced	−P-S-Zn	5,173*	4,693*	4,933*	259	265	262
Enhanced	−K-B	5,559*	4,896	5,228	253*	259*	256*
Enhanced	−P-S-Zn-K-B	5,170*	4,675*	4,923*	253*	257*	255*
Enhanced	−N	5,321	4,759*	5,040*	256*	259^‡^	258*
Enhanced	−Population	4,871*	4,606*	4,738*	277*	282*	280*
Enhanced	−Protection	5,388	4,922	5,155	257*	259*	258*

*Values are the average of 13 trials from six environments in Illinois from 2014–2018.*

*^†^“None” in the exception column indicates the control.*

*^‡^Significant at the 0.10 probability level compared to the respective control treatment.*

*Significant at the 0.05 probability level compared to the respective control treatment.

Averaged across the environments and row spacings, P-S-Zn fertility, sidedress N, and plant population had the most prominent effects on KN with significant decreases when omitted from the enhanced control and increases when added to the standard control (Table 6). Plant population had the most significant impact on KW, which responded negatively to increased population and positively to decreased population. Additionally, KW decreased when K-B fertility, sidedress N, or foliar protection were removed from the enhanced control.

Indicated by a higher KN (6.3%), the preplant banded P-S-Zn application increased yield potential compared with the standard control (Table 6). Conversely, the yield responses to K-B fertilizer were generally associated with changes in KW. Positive yield responses to sidedressing N were associated with KN and KW, as both were increased when sidedress N was included in either the standard or enhanced system. The marginal plant population effect on grain yield resulted from contrasting changes in the yield components. Increasing plant population without increasing other crop inputs (i.e., standard system) resulted in a 5.8% increase in KN and a 6.8% reduction in KW. Decreasing plant population in the enhanced system resulted in an 8.5% decrease in KN and a 6.9% increase in KW. Kernel number response to increasing plant population was more significant in narrow rows than in wide rows for both management systems, suggesting that the plants had a heightened ability to maintain kernels per ear at the high plant population when in the narrow rows. Removing foliar protection from the enhanced system reduced KW by 1.5%.

### System Effects

**FIGURE 2:**
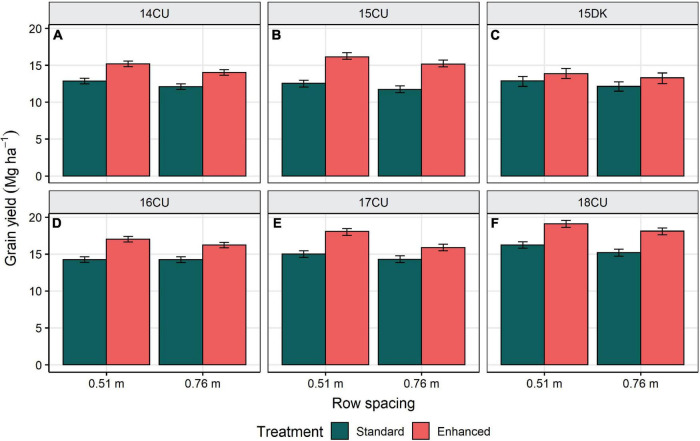
Row spacing influence on grain yield for the standard and enhanced management control treatments at the environments 14CU **(A)**, 15CU **(B)**, 15DK **(C)**, 16CU **(D)**, 17CU **(E)**, and 18CU **(F)**. The bars represent ± 1 SE from the mean. All means are presented as the average of two trials and six replications.

The maize yield gap was estimated as the difference between the standard management control with 0.76 m row spacing, representing typical farming practice, and the enhanced management control with 0.51 m row spacing, representing attainable yield through the implementation of enhanced agronomic management technologies. The average yield gap across the six environments was 3.3 Mg ha^–1^ (25%) and ranged from 1.8 to 4.6 Mg ha^–1^ (15–40%) (*P* < 0.0001) (Table 4 and Figure 2).

The experimental design allows for assessing the additive and synergistic effects from combining the management factors, as portrayed by Ruffo et al. (2015). Estimating the individual yield value of any single management factor can be done with the difference between the standard addition and standard control treatments. Averaged across environments, individual factors that significantly changed yield when added to the standard control in 0.76 m row spacing were P-S-Zn fertility, sidedress N, plant population, and foliar protection, as well as narrowing row spacing to 0.51 m (Table 4). If combinations of factors acted additively in changing yield, summing the individual values for these significant factors gives an additive yield value of 1.7 Mg ha^–1^ (Table 7). However, the actual yield response from combining all the factors was 3.3 Mg ha^–1^ with a 95% *CI* of 3.0–3.7 Mg ha^–1^, which was obtained by calculating the difference between the enhanced control in 0.51 m row spacing and the standard control in 0.76 m row spacing (i.e., the yield gap). Because the lower limit of 3.0 Mg ha^–1^ is markedly higher than the summation of all the individual factor contributions, 1.7 Mg ha^–1^, these management factors are acting synergistically in their effects on grain yield when combined. A significant synergistic effect was also observed within either row spacing and when averaged across the row spacings.

**TABLE 7:** Comparisons between the overall yield difference between the enhanced (Enh) and standard (Std) control treatments (shown as the mean and 95% *CI*; μ_Enh_−μ_Std_) and the summation of the additional yield values provided by each added treatment to the Std control (i.e., Std + P-S-Zn, Std + P-K, Std + N, Std + Population, Std + Foliar protection).

	Row Spacing			
Treatment	0.51 m	0.76 m	Average	0.51 Enh vs. 0.76 Std
	**—————————————————————————————————— Mg ha^–1^ —————————————————————————————————**			
μ_Enh_−μ_Std_	2.7 (2.3–3.0)	2.1 (1.8–3.0)	2.4 (2.2–2.6)	3.3 (3.0–3.7)
∑(*Y*_ + *FACTOR*_−*Y*_*Std*_)‡	1.4	1.1	1.3	1.7

*The additional yield value provided by each treatment was calculated as the difference between the + factor and the Std control yield when significant.*

*^‡^∑[(Y_+P−S−Zn_ − Y_Std_) + (Y_+K−B_ − Y_Std_) + (Y_+N_ − Y_Std_) + (Y_+Pop_ − Y_Std_) + (Y_+Foliar_ − Y_Std_)],*

*∑[(Y_+P − S − Zn_ − Y_Std_) + (Y_+K − B_ − Y_Std_) + (Y_+N_ − Y_Std_) + (Y_+Pop_ − Y_Std_) + (Y_+Foliar_ − Y_Std_) + (Y_.51Std_ − Y_.76Std_)].*

## Discussion

This research estimates the yield gap present in the non-irrigated conditions of Illinois, United States, with contemporary maize hybrids. Across six environments, the combined factors of narrower row spacing, increased plant population, season-long crop nutrition, and foliar protection increased average yield by 25% (3.3 Mg ha^–1^) compared with the standard management practices (Table 4). This data suggests that the maize yield gap can be significantly lessened with narrower row spacing and other enhanced agronomic management technologies. Because consistent yield responses to combining management factors were observed in all the environments of this study (Figure 2), it is expected that the apparent yield gap and management effects on yield would be similar in other highly-productive regions of the United States Corn Belt. However, the current maize yield may be relatively close to the potential yield in the water-limited regions of the Western United States Corn Belt with a higher dependency on irrigation than other management factors for achieving greater yields (Grassini et al., 2011; Balboa et al., 2019).

Notably, all the management factors were necessary for the higher maize yield achieved in the enhanced system, as demonstrated by the yield reductions when any one factor was removed from the system, and no single factor could account for the entirety of the observed yield gap (Tables 4, 5). Narrow row spacing and the combination of P-S-Zn and K-B fertilizer applications resulted in the most significant yield increases of 7.8% and up to 8.4%, respectively, when combined with all other enhanced factors. The magnitude of yield response to the applied fertilizer was not necessarily indicative of the existing soil fertility levels. Banding P-S-Zn containing fertilizer was essential in determining yield potential through impacts on KN (Table 6). The nutrients P, S, and Zn are crucial to kernel development, considering their high harvest indices and remobilization to the grain after flowering (Bender et al., 2013). K-B fertilizer helped maintain KW, especially in high plant population and in wide rows when crowding was presumably higher. As the plant population increases, there is greater competition for nutrients, and K plays a vital role in stalk strength and harvestability (Bohling, 1975; Maria and Farina, 1984). The B supplied with K fertilizer may have aided increases in KW because of its significant translocations during pollination, especially in the presence of potassium fertilizer (Woodruf et al., 1987; Bender et al., 2013).

Sidedress N applications can increase N availability to the crop during pollination and grain-fill but do not always result in greater yield, especially when the initial N levels are adequate and N deficiency is not present before sidedress (Binder et al., 2000). Supplemental sidedress N could have been less impactful in the enhanced management system because of the additional 34 kg N ha^–1^ supplied with the banded P-S-Zn fertilizer. However, with a higher plant density, such as in the enhanced management system, a lower tolerance to low-N conditions and a higher response to sidedressed N applications is expected (Boomsma et al., 2009).

An inverse relationship was observed between KN and KW in response to increasing the plant population, resulting in marginal changes in the grain yield (Tables 4, 6). This inverse relationship between yield components is called “yield component compensation” and is a vital developmental process of plants for maintaining yield when faced with stresses, such as intraspecific competition (Adams, 1967). When reducing plant population in the enhanced system, the reduction in KN per area was proportionally less than the reduction in plant population from 109,000 to 79,000 plants ha^–1^, indicating more kernels developed per plant when at the lower population and all other enhanced factors remained in the system. Increasing plant population heightens intraspecific competition for limiting resources (Boomsma et al., 2009) and limits the ability of plants to obtain limiting resources due to reduced root biomass (Jiang et al., 2013; Bernhard and Below, 2020). Density-induced stress was likely partially alleviated with the applied fertilizer in the enhanced management system leading to a greater tolerance of the high plant population.

The narrow row spacing increased yield primarily through higher KN and was especially important at maintaining kernel set at the higher plant population (Table 6). Narrow rows are more commonly conducive to higher maize yields north of latitude 43°N, mainly because increased light interception from narrowing row spacing becomes more critical in shorter growing seasons (Lee, 2006). However, consistent yield increases from narrowing row spacing were observed across the environments of this study (Figure 1), all of which are south of latitude 43°N. Notably, the response to the narrower row spacing was most significant in the enhanced management system and lessened when reducing the plant population to 79,000 plant ha^–1^ (Table 4). While more favorable responses to narrowing row spacing would be expected in northern latitudes (Lee, 2006), reduced row spacing may be optimal in the central United States when yield potential or plant densities are higher. Reducing row spacing (<0.76 m) increases root biomass and the ability of plants to obtain limiting resources, allowing for greater optimal plant densities (Bernhard and Below, 2020). Increasing plant population beyond the United States average can increase grain yield with modern maize hybrids when other management factors are optimized to mitigate stresses (Table 4). However, other yield components, such as kernels per ear and kernel weight, cannot be maintained at higher densities when resources are limited. Greater planting densities necessitate enhanced management of other, potentially limiting, resources and are better suited for narrower row arrangements.

Foliar fungicides can be effective at increasing maize yield (Ruffo et al., 2015; Vitantonio-Mazzini et al., 2020), and growers more commonly utilize fungicides when the planting density and nutrient availability are higher (Vitantonio-Mazzini et al., 2020). In the environments where significant fungal leaf disease was absent, any observed yield response to strobilurin fungicide (Table 5) was likely due to their “greening effect,” which can maximize grain-filling duration by extending photosynthetic capacity later in the season (Bartlett et al., 2002). Strobilurin fungicide was especially effective in the enhanced system, as the grain-filling rate and final kernel weight are typically depressed under high plant densities (Wei et al., 2019). Greater impacts of fungicide applications may have been observed if consistently more disease pressure was present across the trials, as foliar fungal diseases reduce the photosynthetic area and stalk strength of plants (Dodd, 1977; Wise and Mueller, 2011), resulting in reduced yields.

This work demonstrates that the yield reduction resulting from omitting an agronomic factor from the enhanced system was generally more significant than the yield increase from adding that factor to the standard control (Table 4). Additionally, the combination of enhanced management factors had a synergistic effect on the grain yield in this study. The yield increase from combining all factors in the enhanced system was more significant than the additive response from each management factor applied individually (Table 7). Therefore, when managing maize for greater yields, a comprehensive systems approach will often increase yield more than enhancing any one management factor alone. This research confirms that KN is the yield component most associated with changes in grain yield and is highly impacted by planting population and the availability of nutrients. Thus, closing the maize yield gap will require a systems approach to agronomic management, including better crop nutrition and optimization of spatial plant density.

## Data Availability Statement

The raw data supporting the conclusions of this article will be made available by the authors, without undue reservation.

## Author Contributions

FB guided all facets of this research. TB and EW organized the design and implementation of experiments. EW analyzed the data and wrote the results. EW and FB prepared the manuscript for publication. All authors contributed to the article and approved the submitted version.

## Conflict of Interest

The authors declare that the research was conducted in the absence of any commercial or financial relationships that could be construed as a potential conflict of interest.

## Publisher’s Note

All claims expressed in this article are solely those of the authors and do not necessarily represent those of their affiliated organizations, or those of the publisher, the editors and the reviewers. Any product that may be evaluated in this article, or claim that may be made by its manufacturer, is not guaranteed or endorsed by the publisher.
