# Supplementary material for: Managing Density Stress to Close the Maize Yield Gap
Source: Front Plant Sci. 2021 Dec 15;12:767465. doi: 10.3389/fpls.2021.767465 (PMC8714944; doi:10.3389/fpls.2021.767465)
Supplement: Supplementary file 1 [file Data_Sheet_1.docx]

Supplementary Material

**Supplementary Table 1.** List of trials conducted at Champaign and DeKalb, IL from 2014 to 2015, their planting dates, hybrid planted, and preplant soil test results. Mineral nutrient values were obtained via Mehlich 3-extraction-based method.

| Site-year | Planting date | Maize hybrid | Foliar protection product† | CEC | pH | OM | P | K | Ca | Mg | S | Zn | B |
| --- | --- | --- | --- | --- | --- | --- | --- | --- | --- | --- | --- | --- | --- |
| **2014** |  |  |  | meq 100g^-1^ |  | % | --------------------------- ppm --------------------------- | | | | | | |
| Champaign | 03 June | DKC63-33RIB | Headline AMP | 18.7 | 5.2 | 3.4 | 46 | 138 | 1850 | 366 | 9 | 1.0 | 0.3 |
| Champaign | 06 June | G09E98-3000GT | Headline AMP | 18.1 | 5.5 | 3.3 | 43 | 144 | 1861 | 432 | 8 | 1.1 | 0.4 |
| Champaign | 03 June | 6065SSRIB | Headline AMP | 17.0 | 5.4 | 3.4 | 36 | 118 | 1784 | 362 | 9 | 1.1 | 0.3 |
| **2015** |  |  |  |  |  |  |  |  |  |  |  |  |  |
| Champaign | 07 May | G12J11-3111A | Quilt Excel | 23.3 | 6.1 | 4.2 | 13 | 122 | 2853 | 615 | - | - | - |
| Champaign | 13 May | 70878VT2P | Headline AMP | 22.9 | 5.6 | 3.8 | 11 | 101 | 2452 | 522 | - | - | - |
| DeKalb | 22 May | G06N80-3111 | Quilt Excel | 27.3 | 6.7 | 6.5 | 42 | 172 | 3567 | 897 | 8 | 4.1 | - |
| **2016** |  |  |  |  |  |  |  |  |  |  |  |  |  |
| Champaign | 22 April | DKC63-60RIB | Headline AMP | 18.2 | 5.8 | 3.3 | 32 | 113 | 2088 | 466 | 9 | 1.5 | 0.3 |
| Champaign | 22 April | G14R38-3000GT | Trivapro + Warrior II | 19.3 | 6.0 | 3.3 | 39 | 137 | 2269 | 477 | 8 | 1.8 | 0.3 |
| Champaign | 19 April | 6265SSRIB | Headline AMP | 18.6 | 6.3 | 3.3 | 31 | 131 | 2304 | 518 | 7 | 1.6 | 0.4 |
| **2017** |  |  |  |  |  |  |  |  |  |  |  |  |  |
| Champaign | 18 May | DKC63-60RIB | Headline AMP | 21.2 | 5.4 | 3.8 | 16 | 105 | 2309 | 408 | 9 | 1.1 | 0.4 |
| Champaign | 18 May | G10T63-3122 | Trivapro + Warrior II | 20.2 | 5.6 | 3.9 | 13 | 95 | 2334 | 415 | 8 | 1.3 | 0.4 |
| **2018** |  |  |  |  |  |  |  |  |  |  |  |  |  |
| Champaign | 26 May | DKC63-60RIB | Headline AMP | 20.0 | 6.3 | 3.6 | 40 | 131 | 2566 | 531 | 9 | 2.0 | 0.4 |
| Champaign | 26 May | G11F16-3111A | Trivapro + Warrior II | 19.6 | 6.4 | 3.4 | 35 | 124 | 2488 | 533 | 8 | 2.0 | 0.5 |

**†** Foliar protection products were applied at their labeled rate at the VT/R1 growth stage.

**Supplementary Table 2.** Average monthly temperature (Temp) and precipitation (Precip) for Champaing-Ubrana, IL (CU) from 1014-2018 and DeKalb, IL in 2015. Values in parentheses represents departure from the normal monthly Temp or Precip. Data was obtained from the Illinois State Water Survey.

| Month | 14CU | | 15CU | | 15DK | | 16CU | | 17CU | | 18CU | |
| --- | --- | --- | --- | --- | --- | --- | --- | --- | --- | --- | --- | --- |
|  | Precip | Temp | Precip | Temp | Precip | Temp | Precip | Temp | Precip | Temp | Precip | Temp |
|  | mm | °C | mm | °C | mm | °C | mm | °C | mm | °C | mm | °C |
| April | 103 (6) | 11.6 (0.1) | 95 (-2) | 12.6 (1.1) | 96 (17) | 10.1 (0.9) | 97 (0) | 11.7 (0.2) | 157 (60) | 13.9 (2.4) | 63 (-34) | 7.8 (-3.7) |
| May | 105 (-17) | 17.8 (0.4) | 154 (31) | 18.9 (1.5) | 142 (41) | 16.4 (0.6) | 120 (-3) | 16.4 (-1.0) | 143 (21) | 16.3 (-1.1) | 105 (-17) | 22.3 (4.9) |
| June | 229 (113) | 22.9 (-2.6) | 229 (113) | 22.5 (0.0) | 171 (73) | 20.5 (-0.6) | 144 (28) | 23.3 (0.8) | 65 (-51) | 22.8 (0.3) | 185 (70) | 24.0 (1.5) |
| July | 204 (96) | 21.4 (-2.6) | 106 (-2) | 23.3 (-0.7) | 93 (8) | 21.7 (-0.5) | 112 (4) | 23.7 (-0.3) | 57 (-51) | 25.2 (1.2) | 81 (-27) | 23.7 (-0.3) |
| August | 36 (-50) | 23.0 (-0.1) | 81 (-6) | 22.5 (-0.6) | 73 (-15) | 20.4 (-0.5) | 104 (18) | 24.0 (1.0) | 56 (-30) | 22.0 (-1.1) | 101 (15) | 24.0 (0.9) |
| September | 89 (9) | 18.1 (-1.2) | 164 (83) | 20.9 (1.5) | 75 (-4) | 19.3 (2.2) | 141 (60) | 21.1 (1.7) | 21 (-59) | 20.7 (1.4) | 118 (38) | 21.5 (2.2) |
| October | 125 (38) | 12.2 (-0.4) | 39 (-47) | 13.5 (0.9) | 57 (16) | 11.8 (1.1) | 44 (-42) | 15.1 (2.4) | 161 (74) | 14.0 (1.4) | 55 (-31) | 12.5 (-0.2) |

**Supplementary Table 3.** Kernel number response to fourteen management systems for six environments in Illinois from 2014-2018 and the average of environments. Values are the average of two row spacings (0.76 m and 0.51 m) and multiple trials within each environment.

| Treatment | |  | Environment | | | | | | Mean |
| --- | --- | --- | --- | --- | --- | --- | --- | --- | --- |
| System | Exception |  | 14CU | 15CU | 15DK | 16CU | 17CU | 18CU |  |
|  |  |  | ------------------------------------------- kernels m^-2^ ------------------------------------------- | | | | | | |
| Standard | None† |  | 3724 | 3840 | 4666 | 4415 | 4602 | 4804 | 4342 |
| Standard | +P-S-Zn |  | 3897* | 4356* | 4924* | 4580* | 4832* | 4983‡ | 4595* |
| Standard | +K-B |  | 3825 | 3799 | 4674 | 4386 | 4503 | 4869 | 4343 |
| Standard | +P-S-Zn-K-B |  | 3917* | 4382* | 4806 | 4472 | 4724 | 4952 | 4542* |
| Standard | +N |  | 3861‡ | 4070* | 4754 | 4585* | 4695 | 4931 | 4483* |
| Standard | +Population |  | 4020* | 3833 | 4754 | 4701* | 4769‡ | 5456* | 4589* |
| Standard | +Protection |  | 3863‡ | 3792 | 4663 | 4569* | 4538 | 4903 | 4388 |
|  |  |  |  |  |  |  |  |  |  |
| Enhanced | None |  | 4398 | 5037 | 5317 | 5267 | 5419 | 5640 | 5180 |
| Enhanced | -P-S-Zn |  | 4257‡ | 4547* | 5087 | 5067* | 5024* | 5618 | 4933* |
| Enhanced | -K-B |  | 4387 | 5066 | 5762* | 5213 | 5241* | 5697 | 5228 |
| Enhanced | -P-S-Zn-K-B |  | 4289 | 4514* | 5108 | 5130‡ | 4916* | 5580 | 4923* |
| Enhanced | -N |  | 4373 | 4816* | 4962* | 5123‡ | 5254‡ | 5713 | 5040* |
| Enhanced | -Population |  | 3987* | 4813* | 5152 | 4593* | 4873* | 5014* | 4738* |
| Enhanced | -Protection |  | 4283 | 5139 | 5428 | 5215 | 5261‡ | 5602 | 5155 |

†“None” in the exception column indicates the control.

‡Significant at the 0.10 probability level compared to the respective control treatment.

*Significant at the 0.05 probability level compared to the respective control treatment.

**Supplementary Table 4.** Kernel weight (expressed at 0% moisture content) response to fourteen management systems for six environments in Illinois from 2014-2018 and the average of environments. Values are the average of two row spacings (0.76 m and 0.51 m) and multiple trials within each environment.

| Treatment | |  | Environment | | | | | | Mean |
| --- | --- | --- | --- | --- | --- | --- | --- | --- | --- |
| System | Exception |  | 14CU | 15CU | 15DK | 16CU | 17CU | 18CU |  |
|  |  |  | --------------------------------------- mg kernel^-1^ --------------------------------------- | | | | | | |
| Standard | None† |  | 286 | 260 | 226 | 274 | 269 | 277 | 265 |
| Standard | +P-S-Zn |  | 285 | 261 | 218* | 274 | 275‡ | 274 | 264 |
| Standard | +K-B |  | 286 | 259 | 229 | 273 | 274 | 278 | 267 |
| Standard | +P-S-Zn-K-B |  | 289 | 269* | 229 | 277 | 278* | 284* | 271* |
| Standard | +N |  | 287 | 278* | 229 | 276 | 269 | 283‡ | 270* |
| Standard | +Population |  | 271* | 238* | 212* | 257* | 255* | 251* | 247* |
| Standard | +Protection |  | 293* | 256 | 226 | 275 | 268 | 277 | 266 |
|  |  |  |  |  |  |  |  |  |  |
| Enhanced | None |  | 282 | 263 | 214 | 268 | 265 | 280 | 262 |
| Enhanced | -P-S-Zn |  | 282 | 262 | 216 | 271 | 263 | 279 | 262 |
| Enhanced | -K-B |  | 275* | 266 | 207 | 261* | 259* | 268* | 256* |
| Enhanced | -P-S-Zn-K-B |  | 278 | 255* | 210 | 265 | 254* | 268* | 255* |
| Enhanced | -N |  | 278‡ | 248* | 215 | 268 | 264 | 274* | 258* |
| Enhanced | -Population |  | 295* | 292* | 229* | 283* | 285* | 294* | 280* |
| Enhanced | -Protection |  | 273* | 262 | 209 | 262* | 265 | 277 | 258* |

†“None” in the exception column indicates the control.

‡Significant at the 0.10 probability level compared to the respective control treatment.

*Significant at the 0.05 probability level compared to the respective control treatment.
